# Key Decision-Making Factors in Pediatric Microtia Repair

**DOI:** 10.1177/00034894241304935

**Published:** 2024-12-11

**Authors:** Maya Guhan, Grace Anand, Yi-Chun Liu

**Affiliations:** 1 Baylor College of Medicine, Houston, TX, USA; 2 Department of Otolaryngology, Texas Children’s Hospital, Houston, TX, USA

**Keywords:** pediatric hearing loss, pediatric ear malformations, microtia, ear, hearing, otology, otolaryngology

## Introduction

Microtia, a congenital ear malformation ranging from mild abnormalities to complete absence, presents complex surgical decisions for patients and their families. Surgical options include autologous reconstruction (AR) using rib cartilage, porous polyethylene (PPE) implants, and osseointegrated (OI) implants. The AR method uses rib cartilage to create a 3-dimensional (3D) ear framework, typically when the child reaches 9 to 10 years old and requires a 2-part surgery to complete the reconstruction. Despite its impact, there is limited research elucidating the factors affecting decision-making in both parents and children regarding surgery timing, method of reconstruction, and the perceived versus actual psychosocial impact of ear reconstruction. This commentary asserts the need for additional research to improve shared decision-making between parents and children in microtia repair.

In the realm of microtia reconstruction, the choice of reconstruction method stands as a pivotal decision. Leto Barone et al^1^ compared the AR and PPE implantation approaches and found that 70.6% of the AR patients experienced complications such as contour dissatisfaction, and 40% of PPE recipients faced issues like ear asymmetry and wound healing. Notably, PPE recipients reported higher satisfaction with cosmetic outcomes. Botma et al’s^2^ study revealed a shift toward rib cartilage construction, with 69.5% of surgically treated patients opting for it. This underscores the lack of consensus regarding the preferred method. Hence, individualized discussions, encompassing factors like complication rates and age appropriateness, are crucial for informed decision-making in microtia reconstruction.

Psychosocial concerns drive decision-making in microtia repair, with parents fearing the emotional burden their children may face due to altered physical appearance. Early reconstruction aims to mitigate potential psychosocial challenges, as children with congenital malformations are more likely to experience decreased self-esteem, increased anxiety, and social withdrawal. However, long-term studies reveal variable impacts on self-esteem and social functioning.^3^ Further research is imperative to understand the change in psychosocial status after reconstruction, the factors motivating parents to consider reconstruction, and how to improve shared decision-making between parents and children.

Due to the limited literature on microtia decision-making, we sought to assess decision-making in other craniofacial abnormalities, most notably cleft palate, to assess what key decision-making factors can also be applied to microtia reconstruction. While microtia reconstruction is primarily performed for esthetic reasons, cleft palate has both a functional and esthetic component, and we sought to identify differences and similarities.

Within the cleft palate literature, several recurrent themes emerge: a lack of decision-making by pediatric patients due to their age, a lack of awareness about cleft palate and the need for surgery, and parental influence swaying adolescents’ opinions to obtain assent.^4^ These themes are mirrored in the microtia decision-making literature as parents likely have difficulty comprehending the complexity of the microtia surgery when faced with the option of reconstruction for their children.

Contrary to common assumptions, research suggests that even children as young as 7 can express treatment preferences, with 14-year-old demonstrating decision-making abilities comparable to adults.^5^ Parents typically provide informed consent, but children should also assent to treatment decisions, which can begin as early as age 7 years. However, evidence remains lacking regarding effective strategies to empower pediatric patients as active participants in the decision-making process.

A review of interventions targeting pediatric patient decision-making interventions found that a majority solely targeted parents, indicating a need for more balanced approaches to engage both parents and patients.^5^ When it comes to decision-making in microtia reconstruction, pediatric patients must be given level-appropriate information about their condition. This ensures that patients can express treatment preferences without the sway of their parents and participate in shared decision-making to improve long-term outcomes and overall satisfaction with the procedure. In any case, shared decision-making should be the focus of the patient-parent-physician interactions.

Motivations for surgery among cleft palate patients often center around expectations for improved appearance and self-esteem.^4^ Similarly, pediatric patients with microtia are likely motivated by the desire to enhance their appearance, considering the functional and cosmetic benefits of microtia repair. Recognizing and understanding the patient’s motivation for surgery will ensure that the patient’s expectations match the actual cosmetic end outcome of the surgery. In addition, parents may feel a moral obligation to choose treatments that normalize their child’s appearance, such as surgery, particularly in the context of craniofacial abnormalities. Recognizing parents’ motivations as “moral” obligations rather than social can help providers assist parents in considering all options, the input of their children, and their motivations for choosing “normalizing treatments.”

## Conclusion

Current microtia literature underscores the need for age-appropriate information dissemination and balanced decision-making in microtia repair. Specifically, parents’ concerns about bullying and ostracizing and the need to “normalize” their child should be balanced with the intention of the child to facilitate shared decision-making. Moving forward, research should be conducted to explore ways to ensure shared decision-making among pediatric patients interested in microtia repair.

## References

[1] Leto Barone AA Harris TGW Schroeder MJ , et al Influential factors when considering reconstruction and post-operative outcomes: a survey of microtia patients and parents. J Plast Reconstr Aesthet Surg. 2021;74(7):1633-1701. 10.1016/j.bjps.2020.12.064 33422496

[2] Botma M Aymat A Gault D Albert DM. Rib graft reconstruction versus osseointegrated prosthesis for microtia: a significant change in patient preference. Clin Otolaryngol Allied Sci. 2001;26(4):274-277. 11559335 10.1046/j.1365-2273.2001.00454.x

[3] Horlock N Vogelin E Bradbury ET Grobbelaar AO Gault DT. Psychosocial outcome of patients after ear reconstruction: a retrospective study of 62 patients. Ann Plast Surg. 2005;54(5):517-524. 15838214 10.1097/01.sap.0000155284.96308.32

[4] Matsunaka E Kumagai Y Ike M Takano S Kogo M. Decision-making process to undergo surgery among adolescent patients with cleft lip and/or palate. Jpn J Nurs Sci. 2020;17(4):e12342. 10.1111/jjns.12342 32390343

[5] Wyatt KD List B Brinkman WB , et al Shared decision making in pediatrics: a systematic review and meta-analysis. Acad Pediatr. 2015;15(6):573-583. 25983006 10.1016/j.acap.2015.03.011

